# Clinical Applications of B-Flow Ultrasound: A Scoping Review of the Literature

**DOI:** 10.3390/diagnostics13030397

**Published:** 2023-01-21

**Authors:** Amun G. Hofmann, Irene Mlekusch, Georg Wickenhauser, Afshin Assadian, Fadi Taher

**Affiliations:** Department of Vascular and Endovascular Surgery, Klinik Ottakring, 1160 Vienna, Austria

**Keywords:** b-flow, ultrasound, imaging, diagnostics, sonography

## Abstract

Coded excitation ultrasound investigations have the potential to augment the resolution, increase the efficiency, and expand the possibilities of noninvasive diagnostic imaging. B-Flow ultrasound, a type of digitally encoded imaging, was developed more than 20 years ago with the aim to optimize the visualization of blood flow. It has been investigated for a plethora of applications so far. A scoping review regarding its clinical applications was conducted based on a systematic literature research. B-Flow has been investigated in various anatomic locations and pathologies. However, previous research is limited by small sample sizes, the rare occurrence of elaborate study designs, the reliance on subjective reports and qualitative data, as well as several potential biases. While results are in general promising, it should therefore still be considered an emerging technology. Nevertheless, the limitations can be addressed in future research and the potential to expand its applications make B-Flow an interesting candidate for further investigations.

## 1. Introduction

Coded excitation is a technology aimed at pulse compression, i.e., a long-phase-encoded pulse sequence will be compressed into a short, high-amplitude pulse upon reception [1]. It has been applied in radar and communication technology for more than 60 years and was adapted for commercial ultrasound devices around 20 years ago [2,3]. Ultrasound imaging quality is based on resolution and tissue penetration and the two features are in a tradeoff with each other [2]. Coding on transmit with a broadband, temporally elongated pulse and subsequent decoding on receive results in improved signal-to-noise ratio (SNR) through increased peak acoustic power [4]. However, decoding also introduces range side-lobes, which impair imaging quality, and coded excitation is therefore subject to an additional tradeoff between SNR and range side-lobes [4].

Several methods of coded excitation have been investigated for their ability to improve the quality of medical ultrasound studies, especially at high frequencies facilitating ultrasound biomicroscopy [5]. Linear chirp-coded signals combined with annular arrays can lead to improved SNR and depth-of-field in high-frequency ultrasound investigations [6]. The high computational complexity that was historically required and criticized can be reduced by efficient pulse compression methods that maintain the axial resolution and high SNR [7,8]. Chirp-coded ultrasound might be specifically useful in ophthalmology, dermatology, and small-animals imaging [5,6]. Nonlinear frequency modulation and Golay codes have also been shown to improve the SNR [9]. Other codes used in sonographic imaging include white noise, pseudo-random code, or pseudo-chirp [10].

Coded excitation has been specifically investigated to improve sonographic studies in vascular medicine in various ways. The beneficiary effects of coded excitation on color flow imaging applications have been repeatedly discussed [11]. Barker-coded color flow images showed augmented axial resolution and increased penetration [12]. Recently, a new method, the differential compression of Golay-encoded ultrasound, has been reported to reliably facilitate slow-blood-flow imaging in small vessels [13]. Nevertheless, regarding the optimization of blood flow and vasculature imaging through coded excitation, B-Flow arguably had the highest impact in both scientific literature and clinical practice.

B-Flow is a type of digitally encoded ultrasound specifically developed by GE Healthcare (Chicago, IL, USA) for blood flow visualization. Its technical foundation is based on a combination of coded excitation and tissue equalization [1]. Essentially, B-Flow displays blood flow dynamics by digitally subtracting echoes from two or more pulses and amplifying the difference [14]. In stationary tissue, this difference is approximately zero. However, with moving erythrocytes, the derivative between pulse signals equals a nonzero value [14]. B-Flow has been investigated for different applications over the course of the last 20 years. While, historically, duplex ultrasound has been the prime imaging modality for vasculature, B-Flow’s advantages were described to be embodied by its technical foundation. Fewer pulses on a given line combined with high-frequency wideband pulses were discussed to allow for improved axial resolution and high-frame-rate capabilities [1,14,15]. However, B-Flow does not provide information on the velocity or direction of flow [16]. Nevertheless, the result is a more precise image of the vessel and blood flow compared to the duplex ultrasound [14]. Doppler artifacts such as color blooming or aliasing are absent in B-Flow [15].

## 2. Methods

**Figure 1:**
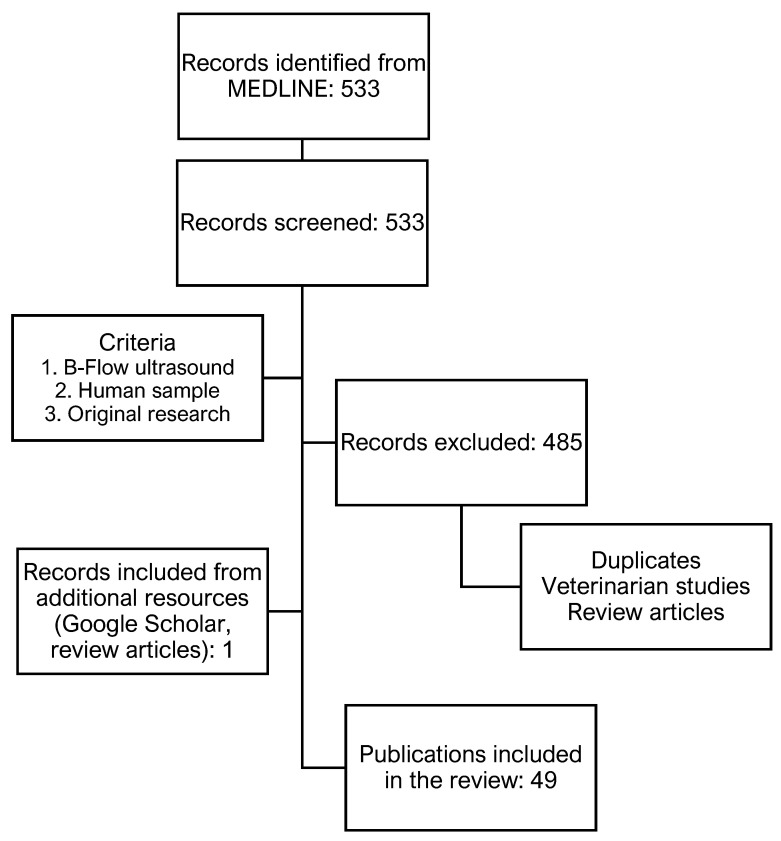
Flow diagram illustrating literature research.

Literature was compiled and filtered based on routine review workflows. MEDLINE was screened using the search queries “b-flow”, “b-flow ultrasound”, and “b-flow imaging” in October and November 2022. This resulted in 533 publications. Initially, duplicates were removed and search results were screened based on title, with a second-layer filter using information provided in the abstracts. Articles were filtered based on the following criteria: (1) application of B-Flow ultrasound, (2) human study population, and (3) original clinical research including case reports and investigator experience reports. Additionally, a complementary literature research strategy using Google Scholar was conducted in November 2022 to validate and supplement the findings. Furthermore, review and summary articles identified through the presented search strategy were screened for potentially otherwise missed publications. The last two steps resulted in the addition of a single publication. In total, the current review is compiled based on the findings of 49 original articles. Figure 1 illustrates the conducted literature research.

### 2.1. Abdominal Ultrasound

Historically, the examination of the vasculature of the liver constitutes one of the first applications of B-Flow, such as the exploratory investigation and anatomic description of hepatic arteries in chronic liver disease [17]. In hepatocellular carcinoma, it was praised for its ability to depict intratumoral vessels [18], especially if complemented by contrast enhancers [19]. B-Flow not only provided more accurate results regarding hepatic tumor vascularization, contrast enhancement was also detectable for longer when compared to other types of sonography, facilitating improved examination of tumor margin vascularization [20]. The quantitative data is complemented by qualitative experiences that describe B-Flow to be more reliable compared to the duplex ultrasound for the investigation of hepatic vessels due to a reduction in artifacts that can otherwise lead to erroneous diagnoses [21]. A very recent case report of a patient diagnosed with hepatic sclerosed hemangioma discussed the advantages of a multimodal ultrasound-based imaging algorithm that includes contrast-enhanced ultrasound, ultrasound elastography, as well as B-Flow [22]. This reflects a recurring pattern regarding research on B-Flow, i.e., its ability to improve diagnostics when being combined with other imaging modalities.

Investigations regarding B-Flow in hepatology are complemented by an investigator experience report covering its applicability for abdominal ultrasounds in general. Apart from hepatic vessels, it is discussed to be well-suited for imaging studies of visceral arteries and the aorta. The discussed advantages of B-Flow focus on its user-friendliness, simplicity, and resolution, which are valuable as a complement to acquired Doppler images [23].

**Figure 2:**
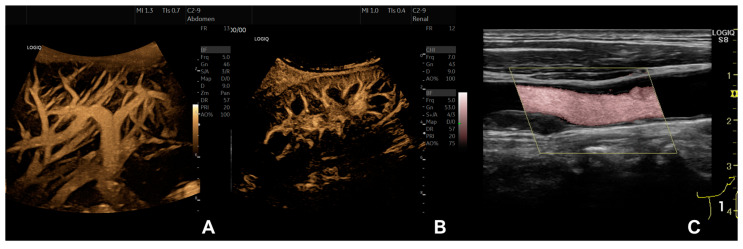
Examples of B-Flow ultrasound imaging of the liver (**A**), kidney (**B**), and carotid artery (**C**). Used with permission of GE Healthcare.

**Figure 3:**
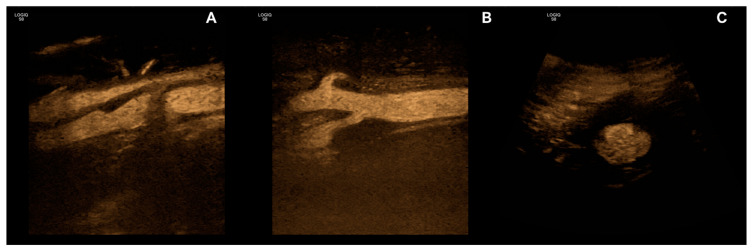
Examples of vascular pathologies visualized with B-Flow, carotid artery stenosis with calcified (**A**) and soft plaque (**B**) as well as abdominal aortic aneurysm with mural thrombus (**C**).

Renal blood flow can also be sufficiently visualized and examined by B-Flow. Compared to the duplex ultrasound, it proved to be more accurate when estimating renal artery stenosis while being validated against findings from digital subtraction angiographies [24]. B-Flow is also well-suited to study the vasculature of kidney transplants, especially as a complement to routine ultrasound follow-up protocols. This has been both investigated and shown in adult [25] and pediatric [26] post-transplant patients. Examples of B-Flow images of the liver and kidney vasculature are depicted in Figure 2A,B, while Figure 3C shows an abdominal aortic aneurysm.

### 2.2. Obstetrics and Gynecology

Several clinical studies investigated B-Flow imaging for potential applications within obstetrics and gynecology. Most frequently, the research is focused on utilizing the technology in perinatology in general and fetal echocardiography in general. Compared to other specialties, the use of B-Flow in obstetrics and gynecology is frequently investigated in the form of 4-dimensional ultrasound with B-flow imaging and spatiotemporal image correlation. Spatiotemporal image correlation is a post-acquisition technology that produces a dynamic 3-dimensional image of the fetal heart that allows multiplanar examination. Commonly, 3- or 4-dimensional sonography and spatiotemporal image correlation are paired with a conventional duplex as the state-of-the-art technology to investigate congenital disorders of the heart in prenatal settings [27]. Color Doppler echocardiography can detect the fetal ductus venosus reliably. However, its accuracy increases with the diameter of the ductus venosus and is therefore dependent on gestational age. The use of 4-dimensional echocardiography with B-Flow and spatiotemporal image correlation was found not only to also offer accurate ductus venosus imaging but to facilitate higher study quality at 18–29 weeks of gestation than the regular duplex ultrasound [28]. Similarly, a small study (n = 12) investigated 4-dimensional echocardiography with B-Flow and spatiotemporal image correlation for the visualization of pulmonary atresia and ventricular septal defect in fetuses, which proved to be more reliable than conventional 2-dimensional ultrasound or duplex imaging [29]. Similar findings in 7 fetuses were reported for the prenatal identification of isolated total anomalous pulmonary venous connection. However, the study is also remarkable considering it is one of the larger B-Flow-backed investigations, with a total of 1040 studied fetuses included [30]. A case report further suggested the introduction of a corresponding sign, the *starfish sign*, that upon revelation can indicate total anomalous pulmonary venous return [31]. Subsequently, the technology was applied in the investigation of interrupted aortic arches, yet again indicating the better depiction of the pathology compared to standard 2-dimensional ultrasound in a cohort of 22 fetuses [32]. Apart from congenital cardiac conditions, B-Flow and 4-dimensional echocardiography with B-Flow and spatiotemporal image correlation was additionally praised for the prenatal visualization of small vessels such as pulmonary veins that are characterized by low flow velocity [33].

An investigator experience further discusses the advantageous visualization of complex flow patterns in small vessels that B-Flow produces. Combined with the fact that it is angle-independent and undisturbed by information produced through B-Mode (since they both work on the same spatial resolution and frame-rate), B-Flow is reported to be well-suited for umbilical blood flow imaging, as well as for investigation of the neonatal vascular system [34]. While these reports are now more than 20 years old, recently a cohort of 34 neonates underwent B-Flow sonography for investigation of the basal cerebral arteries, producing evidence that the technology is indeed at least noninferior and potentially superior for basal cerebral artery imaging compared to other types of ultrasound [35].

Apart from fetuses and neonates, B-Flow was also used to investigate the placenta in 36 pregnant women, including 17 high-risk pregnancies. In a comparably elaborate study design with two independent blinded investigators, B-Flow and color Doppler were compared to each other. While B-Flow was able to visualize significantly more small vessels, both horizontal and vertical, than the color Doppler, the authors noted that the potential capturing of artifacts requires modifications during image acquisition, such as decreasing the pulse repetition interval to suppress flash artifacts [36].

Outside of obstetrics, B-Flow was evaluated as an alternative imaging modality in hysterosalpingo-contrast sonography. In one of the larger studies concerned with the technology, 160 women underwent an ultrasound, first with saline and air and subsequently with pharmaceutical contrast enhancement. Especially when using saline and air, B-Flow resulted in improved visualization of the middle and distal parts of the fallopian tubes compared to the grayscale ultrasound [37].

### 2.3. Vascular Medicine

#### 2.3.1. Extracranial Arteries

Early investigations of B-Flow focused on the imaging of atherosclerotic carotid artery stenosis. In the early 2000s, the first investigations reported favorable results regarding the visualization of flow patterns and disease extent. However, these studies were conducted as feasibility trials with less than 30 patients [38,39]. Subsequently, B-Flow was described to facilitate high spatial resolution at a high frame rate that resulted in satisfactory visualization of atherosclerotic lesions and reliable estimation of stenosis degree compared to other ultrasound-based technologies such as power Doppler imaging or duplex sonography [40,41]. This extends to pre-occlusions and occlusions of the internal carotid artery, were B-Flow was again discussed to result in more reliable diagnosis than color coded Doppler and power Doppler [42]. B-Flow studies were also reported to correlate well with computed tomography angiography and magnetic resonance angiography findings for the evaluation of plaque morphology and lesion extent, especially when being applied to 3-dimensional ultrasound investigations [43,44]. Speckle reduction imaging and 3D postprocessing have further resulted in reduced flow artifacts, which is additionally beneficial in postinterventional imaging studies after carotid artery stenting [41]. B-Flow was also reported to have a high correlation with digital subtraction angiography results and was simultaneously praised for superior planimetric accuracy compared to power Doppler imaging [45]. However, while the imaging modality was praised for its low interobserver variability, the measured parameters were also discussed to be inefficient in the evaluation of internal carotid artery stenosis compared to standard duplex ultrasound in an early investigation [46]. Nevertheless, it should be stated that these results essentially all stem from pilot studies with sample sizes commonly below 50 patients. The largest cohort of the initial phase studies included 95 patients, and its authors concluded that, while duplex ultrasound had a slightly higher accuracy in carotid artery stenosis detection, the combined use of both technologies was found to be more accurate than either imaging modality alone [47]. After a spike in publications in the early and mid-2000s, B-Flow-related research regarding the carotid arteries seemingly disappeared until a recent article published in 2020. A cohort of 120 elderly patients underwent bilateral B-Flow imaging of the carotids, which resulted in the successful depiction of plaque morphology [48]. In summary, B-Flow repeatedly proved to be a valuable diagnostic tool to investigate and characterize atherosclerotic lesions of the carotid arteries compared to established imaging technologies.

B-Flow has additionally been investigated for the diagnosis of dissection in the cervical arteries. While magnetic resonance angiography proved to be more accurate with better resolution, and was also used as the reference method, the complementation of other ultrasound modalities with B-Flow could increase detection rates regarding both carotid and vertebral artery dissections. This might be due to the improved visualization of flow within the true and false lumen, of the present thrombi, and of the intramural hematoma [49]. The usefulness of B-Flow for the sonography-based diagnosis of arterial dissections has also been explored in other anatomic locations with similar results [50]. Furthermore, B-Flow has been shown to allow precise studies of fibromuscular dysplasia of the carotid arteries, which is hypothesized to translate well to other anatomic locations [51]. An illustration of a carotid artery using B-Flow is shown in Figure 2C and examples of carotid artery stenosis are depicted in Figure 3A,B.

#### 2.3.2. Vascular Access Care

An early investigation of potential applications for B-Flow was concerned with the assessment of pathologies after arteriovenous fistulas for hemodialysis access such as stenoses, intima flaps, or thrombi. In a qualitative comparative analysis, B-Flow was found to facilitate better imaging regarding the morphology and local degree of stenosis while resulting in less artifacts than duplex sonography or intra-arterial digital subtraction angiography [52]. More recently, 2- and 3-dimensional tomographic ultrasound based on raw data from B-mode, B-Flow, and power Doppler was also studied for its visualization of arteriovenous fistula pathologies. While feasible, the raw data input from B-Flow was discussed to be at a disadvantage compared to the other technologies due to difficulties regarding its reproducibility by less-experienced investigators [53].

#### 2.3.3. Phlebology

Phlebology constitutes an outlier regarding research on B-Flow, since the imaging modality was not investigated per se as for potential indications but rather applied to study venous anatomy and physiology itself. This resulted in an advanced understanding of the mechanism of venous valve closure, characterized by four different phases that valve cusps undergo, and local hemodynamics [54]. Additionally, B-Flow was used in the investigation of CEAP C0S chronic venous disease patients (symptoms with absent pathophysiological explanation). However, while the continuous-wave Doppler was able to capture a reflux on nonaxial veins that might explain symptomatic disease manifestations, B-Flow did not result in any considerable findings [55].

#### 2.3.4. Endovascular Interventions

One of the earliest and largest clinical studies investigating the B-Flow ultrasound included 700 patients in a single-center observational study who underwent sonography examinations after endovascular interventions. Compared to the duplex ultrasound and power Doppler, B-Flow facilitated improved visualization of pseudoaneurysms and exact localization due to the precise demarcation of adjacent soft tissue [56]. Chronologically based on the previous data, a recently published pilot trial shifted from sole visualization to the treatment of pseudoaneurysms by investigating the efficacy of thrombin injections under B-Flow guidance in 21 patients, with favorable results [57]. A recent randomized controlled trial investigated whether the addition of B-Flow to color Doppler imaging can improve the guiding for arterial puncture and catheterization through wounds in patients with large burns. B-Flow not only increased the success rate of arterial puncture but also shortened the catheterization duration and a lowered the incidence of subsequent arterial thrombosis [58].

#### 2.3.5. Peripheral Artery Occlusive Disease

Further applications of B-Flow for pathologies of the peripheral vascular system include peripheral artery occlusive disease. While the benefits of B-Flow compared to duplex ultrasound for imaging of the common femoral artery in the presence of calcified plaques are discussed in a case report [59], it has also been investigated in a cohort of 60 patients affected by peripheral arterial disease or after bypass surgery. The single-center experience, on the one hand, advocates B-Flow for its advantageous visualization of atherosclerotic lesions, while, on the other hand, it discusses limitations to the results in the conclusion, suggesting that its usage should be considered complementary to standard duplex ultrasound [60].

### 2.4. Further Applications

Apart from the main anatomic locations discussed above, B-Flow has been the subject of investigation for certain niche applications and in specialties that are not routinely dependent on ultrasound studies. This, for example, includes the preoperative mapping of potential perforator flaps. It constitutes one of the more recent indications for the use of B-flow sonography after two recently published exploratory trials with 8 and 16 patients, respectively. On the one hand, routine B-Flow sonography is discussed to be a valuable complementary imaging technology within a more extensive mapping algorithm [61]. On the other hand, contrast-enhanced B-Flow ultrasound was found to facilitate complete perforator flap mapping and can thereby increase the quality of and safety of flap harvesting procedures. However, the authors did note that this imaging modality is technically demanding and is more suited for experienced examiners [62].

A case report discussed the use of B-Flow in a patient with suspected giant cell arteritis and the potential advantages for its application in this specific pathology, including factors already mentioned elsewhere, such as the high frame rate with beneficial spatial and contrast resolution, absent angle dependency, as well as less artifacts than duplex sonography [63].

B-Flow studies have also led to the discovery of sonographic signs that are solely visualized by the imaging modality. The so-called *flashlight sign* refers to intraluminal wall-adherent, floating arterial structures in the aorta and periphery, and its presence can simplify their detection in the future [64]. In thyroid papillary cancer, microcalcifications produce a subsequently termed *twinkling sign* that can aid the investigation of suspect thyroid nodules [65]. A similar signal, *twinkling little stars in the night sky*, was described in a recent case report of a patient with acute cholangitis, referring to small, rapidly moving high-echo spots in the portal vein that were confirmed as portal vein gas [66].

Table 1 summarizes the findings of the present review.

## 3. Discussion

### 3.1. Research in Context

The main advantage of coded excitation is an improved signal-to-noise ratio. The specific development of an ultrasound imaging modality that uses digitally encoded excitation to optimize the visualization of blood flow resulted in B-Flow and was a highly regarded technology after its introduction to the market. It was soon reported to augment axial resolution and allow high frame rate capabilities [14,15]. However, even though it was highly touted in a plethora of previous publications, as discussed above, it could not supplant other imaging technologies so far.

Since B-Flow is based on ultrasound, it is subject to the very same limitations of other types of ultrasound, such as routine B-mode imaging. This includes excessive intestinal gas, obesity, or patient adherence/cooperation to imaging studies [67,68]. Similarly, investigator effects apply, including the experience of an examiner, even though solutions for this limitation have been previously proposed [69]. Nevertheless, the same is true for the benefits of ultrasound imaging that span from its lower threshold of availability to rapid application, better cost effectiveness than computed tomography or magnetic resonance imaging, and the absence of ionizing radiation [70]. While the resolution of computed tomography and magnetic resonance imaging is still superior to ultrasound, and therefore B-Flow, the introduction of high-resolution ultrasound (offering B-mode images) might be able to mitigate the differences in resolution [71,72]. We hypothesize that further technological improvements of ultrasound in general will translate to specific modes including B-Flow.

Compared to other types of sonography, the evaluations of B-Flow generally led to satisfying results. Some of the effects and advantages of vascular ultrasound imaging are difficult to capture by objective and quantitative data. However, the repeatedly reported conclusion that B-Flow is more accurate and less limited by artifacts than duplex ultrasound, arguably the prime ultrasound technology for the investigation of the vasculature and blood flow, is unlikely to be based on confounding, bias, and/or randomness alone. Even in most investigations that did not result in a clear advantage of B-Flow, it is commonly reported that its inclusion in imaging algorithms improves diagnostics, if only as a complement to other modalities.

### 3.2. Limitations of Current Evidence

Several limitations are associated with the research on B-Flow imaging. The level of evidence both for the investigation of most applications as well as for the cumulative body of evidence is dominated by case reports, case series, and observational studies that frequently rely on qualitative analyses. The conducted literature research identified only a single outcome-assessing randomized controlled trial. Regardless of the underlying hierarchy of evidence, including GRADE or the standard reference of Guyatt and Sackett [73], most publications would be classified in lower levels. Quantitative data comparing B-Flow to other imaging modalities, for example by measuring agreement and inter-method reliability, or quantitative results on the efficacy of B-Flow itself, such as inter-observer agreement trials, is present for distinct applications only. However, the final validation of new imaging modalities relies on objective metrics.

Inherently linked to the frequently used study designs is the sample size limitation. Most published B-Flow studies are conducted as exploratory pilot studies with associated sample sizes (generally < 50 patients). While both methodologically as well as ethically these sample sizes are legitimate to conduct pilot investigations, they are insufficient to reach conclusive answers and validate imaging technologies. Larger trials do exist and are explicitly highlighted in the sections above, mainly owing to their scarcity.

Finally, another observed major limitation of research on B-Flow ultrasound is the clustering of certain investigators and centers. A large proportion of the effort to investigate potential applications of B-Flow appears to be driven by a comparably small number of investigators, with one group/center contributing 10 out of 49 (20.4%) research publications. While their efforts deserve recognition and appreciation for advancing the field, reproducibility and reliability require the repeated confirmation of results in different settings to compensate for effects, such as investigator bias and learning curves.

Based on the recent European Society of Radiology’s statement on the validation of imaging biomarkers [74] (considering images acquired with B-Flow as imaging biomarkers), requirements include the evaluation of precision (and thereby variability), accuracy, repeatability in various settings (different centers and acquisition protocol configurations), as well as operator influence (intra-operator variability and inter-operator variability). Finally, clinical endpoints should be investigated as a conclusive performance indicator [75]. Currently, B-Flow has yet to achieve the transition from exploratory imaging modality to validated tool for almost all applications based on the absence of such investigations.

### 3.3. Future Perspectives

B-Flow is a useful ultrasound mode that is very likely to improve diagnostics for pathologies regarding the vasculature and hemodynamics. However, apart from the limitations discussed above, a plethora of possible applications still exist in vascular medicine that have yet to be investigated using B-Flow. Since B-Flow was able to visualize venous valve dynamics accurately, it might be well-suited to diagnose and stage chronic venous insufficiency or during treatment with foam sclerotherapy. Especially follow-ups after arterial interventions that require imaging at regular intervals could significantly benefit from B-Flow. In peripheral arterial occlusive disease, B-Flow might be a reasonable option for surveillance after stent placement, as has been done in the carotid arteries. After endovascular aortic repair for abdominal aortic aneurysm, it could be investigated for its ability to detect and classify complications such as endoleaks. Most protocols for these pathologies and treatments are either based on color Doppler imaging, where B-Flow is frequently reported to be more accurate, or computed tomography, which includes other disadvantages such as cumulative radiation exposure.

In conclusion, while B-flow ultrasound is an interesting imaging technology that has been investigated in a plethora of different pathologies, its application has still not exhausted all possibilities, as evidenced by recent efforts to expand its usage. Nevertheless, even though it is a simple and effective addition to other ultrasound studies, its future integration in other areas might be dependent on a combination of perceived usefulness, perceived ease of use, behavioral intention, knowledge, and attitude [76].

## Figures and Tables

**Table 1:** Clinical applications of B-Flow ultrasound based on anatomic location. (PA-VSD: pulmonary atresia and ventricular septal defect, ITAPVC: isolated total anomalous pulmonary venous connection, PAOD: peripheral arterial occlusive disease).

Anatomic Location	Pathologies			
Head/Neck	Carotid artery stenosis Thyroid papillary cancer	Carotid artery stenting Carotid artery occlusion	Carotid/vertebral artery dissection	Refs. [38,39,40,41,42,43,44,45,46,47,48,49,50,51,65]
Abdomen	Abdominal aorta Hepatic tumor vascularization Renal artery stenosis	Visceral arteries Hepatic sclerosed hemangioma	Hepatic arteries Kidney transplants	Refs. [17,18,19,20,21,22,23,24,25,26]
Fetus/Neonate	Fetal ductus venosus Interrupted aortic arch	PA-VSD Umbilical blood flow	ITAPVC Basal cerebral artery	Refs. [28,29,30,31,32,33,34,35]
Female reproductive system	Placenta vascularization	Hysterosalpingo contrast-sonography		Refs. [36,37]
Peripheral vascular system	Arteriovenous fistulas Arterial puncture	(Pseudo-) Aneurysms	PAOD	Refs. [52,53,54,55,56,57,58,59,60]
Other	Perforator flap mapping	Giant cell arteritis		Refs. [61,62,63]
